# Hospital admissions and emergency department visits for people with dementia

**DOI:** 10.1093/qjmed/hcad232

**Published:** 2023-10-09

**Authors:** E Zafeiridi, A McMichael, L O’Hara, P Passmore, B McGuinness

**Affiliations:** Centre for Public Health, Queen’s University, Belfast, UK; Centre for Public Health, Queen’s University, Belfast, UK; Centre for Public Health, Queen’s University, Belfast, UK; Centre for Public Health, Queen’s University, Belfast, UK; Centre for Public Health, Queen’s University, Belfast, UK

## Abstract

**Background:**

Previous studies have suggested that people with dementia (PwD) are more likely to be admitted to hospital, have prolonged hospital stay, or visit an emergency department (ED), compared to people without dementia.

**Aim:**

This study assessed the rates of hospital admissions and ED visits in PwD and investigated the causes and factors predicting this healthcare use. Further, this study assessed survival following hospital admissions and ED visits.

**Design:**

This was a retrospective study with data from 26 875 PwD and 23 961 controls.

**Methods:**

Data from national datasets were extracted for demographic characteristics, transitions to care homes, hospital and ED use and were linked through the Honest Broker Service. PwD were identified through dementia medication and through causes for hospital admissions and death.

**Results:**

Dementia was associated with increased risk of hospital admissions and ED visits, and with lower odds of hospital readmission. Significant predictors for hospital admissions and readmissions in PwD were transitioning to a care home, living in urban areas and being widowed, while female gender and living in less deprived areas reduced the odds of admissions. Older age and living in less deprived areas were associated with lower odds of an ED visit for PwD. In contrast to predictions, mortality rates were lower for PwD following a hospital admission or ED visit.

**Conclusions:**

These findings result in a better understanding of hospital and ED use for PwD. Surprisingly, survival for PwD was prolonged following hospital admissions and ED visits and thus, policies and services enabling these visits are necessary, especially for people who live alone or in rural areas; however, increased primary care and other methods, such as eHealth, could provide equally effective care in order to avoid distress and costs for hospital admissions and ED visits.

## Introduction

Dementia is associated with frequent hospital admissions, prolonged hospital stay and increased risk of emergency department (ED) visits.^1–3^ Common causes for hospital admissions and ED visits in people with dementia (PwD) are urinary tract infections, injuries from falls, behavioural problems and lower respiratory tract infection, such as pneumonia or bronchitis.^4–8^ ED visits and hospital admissions towards the end of life for PwD are likely to result in greater cognitive and physical impairment,^9^^,^^10^ suggesting there is a need to understand the predictors that lead to admissions and ED use in dementia. Past research has shown that male gender, advanced age, living in rural areas and being care dependent are risk factors for hospital admissions in PwD.^3^ Predictors for ED use in PwD include being married, while controversial findings exist on whether males with dementia are at an increased risk of ED visits.^11^ With respect to whether advanced age predicts ED visits in PwD, the evidence is mixed. For instance, some studies have shown that increasing age is associated with greater ED use,^2^ while other evidence showed that younger age is a better predictor of ED visits,^11^ or that age is not associated at all with ED use.^12^ The hospital environment can be distressing for PwD^13^ and their caregivers^14^ and thus, frequent admissions and ED visits may result in increased mortality rates during or 6 months after a hospitalisation in PwD.^7^^,^^15^ In addition, prolonged hospital stays can result in worse healthcare outcomes and worsen cognition.^16^ In many cases, hospital admissions and ED visits can be avoidable,^17^^,^^18^ for example in some cases, infections can be treated without hospitalisation.^6^ Therefore, less hospital admissions and ED visits for PwD could increase their life expectancy and relieve some of the financial strain in healthcare practices. A more in-depth understanding of the causes and factors associated with admissions and ED use could provide useful knowledge about the healthcare use and needs of PwD.

This study explored the rates of hospital admissions and ED visits among PwD and a control group in Northern Ireland. The most common causes for admissions and ED visits and factors associated with hospital and ED use were also explored. Another aim was to assess survival following hospital admissions and ED visits in PwD and controls.

## Methods

### Study population

Similar to our past research,^19^ PwD were identified based on the first date they received a dementia management mediation between 1 January 2010 and 31 December 2016. These data are recorded on the Enhanced Prescribing Database (EPD): this holds information on ∼80–90% of medication prescribed by General Practitioners and dispensed by a pharmacist in Northern Ireland. PwD were also identified through hospital admissions recorded on the Patient Administration System and the Symphony system in Northern Ireland, as well as through causes of death as recorded on death certificates. A total of 25 418 PwD were identified through the EPD. Data for an equal size, age- and gender-matched control group were extracted from the Patient Administration System. Within this control group, 1427 people had a dementia cause of death or hospital admission and were transferred to the dementia group. Demographic data for the gender, age, marital status, area of living and deprivation score, as well as data for hospital admissions and ED visits were extracted and linked through the Honest Broker Service (HBS) in Northern Ireland following approval from their independent committee. Deprivation data were accessed through the Northern Ireland Multiple Deprivation Measure^20^ where higher values indicate less deprived areas on a scale from 1 to 10. This measure used indicators from deprivation measures in the Republic of Ireland and Great Britain. Deprivation was assessed based on different indicators, including income deprivation, employment deprivation, health deprivation and disability, education, skills and training deprivation, access to services, living environment and crime and disorder.^20^ Data for transitions to care homes were extracted through the Business Services Organisation and linked through HBS based on the General Practitioners’ claims for visiting patients in nursing or care home facilities. Similar to our work on transition to care homes for PwD,^21^ care homes in this article refer to long-term care settings, residential care and nursing homes. The date of death was identified through data from death certificates from the General Register Office for people who were deceased between 2010 and 2016.

### Statistical analysis

Descriptive analysis was conducted for both the group of PwD (*N* = 26 875) and the control group (*N* = 23 961). Independent-samples *t*-tests and chi-square analyses were conducted to explore differences in the demographic characteristics and causes for hospital admissions and ED use between the two groups. Binomial regression models were used to explore predictors for admissions and ED visits. Finally, Cox proportional hazard models were conducted to assess survival following hospital admissions and ED visits in PwD and controls.

## Results

### Demographic characteristics

PwD were significantly older (mean = 77.65 years, standard deviation (SD) = 8.43; Table 1) compared to the control group (mean = 76.85 years, SD = 8.38; *P* < 0.001). There were significantly more females (66% PwD, 64% controls; *P* < 0.001) compared to males, and most PwD (69%) and people in the control group (64%) lived in urban areas compared to rural areas (*P* < 0.001). Most PwD (32%) and people in the control group (30%) were married (*P* < 0.001) instead of single, divorced or widowed. Almost 44% of PwD and 9% of people in the control group transitioned to a care home within or prior to the study period. Significantly more PwD (50%) died within this period compared to controls (34%).

**Table 1.** Demographic characteristics for PwD and the control group

	PwD	Control group	*P*-value
*N*	26 845	23 991	
Age (mean, SD)	77.65 (8.43)	76.85 (8.38)	**<0.001** ^a^
Gender (*N*, %)			**0.001** ^b^
Males	9200 (34%)	8560 (36%)	
Females	17 645 (66%)	15 431 (64%)	
Deprivation (mean, SD)	5.67 (2.92)	5.66 (2.84)	0.700^a^
Area of living (*N*, %)			**<0.001** ^b^
Rural	7679 (29%)	8303 (54%)	
Urban	18 419 (69%)	15 313 (64%)	
Marital status (*N*, %)			**<0.001** ^b^
Married	8525 (32%)	7315 (30%)	
Single	1777 (7%)	1531 (6%)	
Divorced	521 (2%)	418 (2%)	
Widowed	6539 (24%)	5647 (24%)	
Transitioned to a care home (*N*, %)	11 739 (44%)	2266 (9%)	**<0.001** ^b^
Number of people with hospital admissions (*N*, %)	23 170 (86%)	19 149 (80%)	**<0.001^b^**
Number of hospitalizations (mean, SD)	4.65 (15.09)	5.13 (27.75)	**0.014^a^**
Hospital stay in days (mean, SD)	18.15 (28.03)	11.54 (19.69)	**<0.001^a^**
Number of people with hospital readmissions (*N*, %)	20 615 (77%)	16 348 (68%)	**<0.001^b^**
Number of people with ED visits (*N*, %)	18 837 (70%)	13 800 (58%)	**<0.001** ^b^
Number of ED visits (mean, SD)	2.56 (3.32)	1.72 (2.60)	**<0.001** ^a^
Number of deaths (*N*, %)	13 514 (50%)	8067 (34%)	**<0.001** ^b^

a Independent-samples *t*-test.

b Chi-square tests.

Bold *P*-value - significance <.001.

Between 2010 and 2016, more PwD (86%) were admitted to a hospital compared to controls (80%, *P* < 0.001; Table 1). PwD were also more likely to be readmitted to a hospital following a first admission than people in the control group (77% and 68%, respectively, *P* < 0.001). PwD had also a lower mean number of hospital admissions (*P* = 0.014) and stayed in hospital for more days (*P* < 0.001) compared to people in the control group. Significantly more PwD (70%) attended an ED compared to people from the control group (58%, *P* < 0.001). The mean number of ED visits was higher for PwD than for the control group (*P* < 0.001).

### Causes for hospital admissions and ED visits

The most common causes for hospital admissions for PwD and the control group are presented in Table 2. PwD were hospitalized more often for a fracture (*P* < 0.001), a urinary tract infection (*P* < 0.001), a lower respiratory tract infection (*P* < 0.001) and syncope and collapse (*P* < 0.001), and less often for cataract (*P* < 0.001) compared to controls. Compared to the control group PwD were also more likely to attend an ED because of a fracture (*P* < 0.001), a head injury (*P* < 0.001), a urinary tract infection (*P* < 0.001), a soft tissue injury (*P* < 0.001) and a lower respiratory infection (*P* < 0.001).

**Table 2.** Most common causes for hospitalizations and ED use

Hospital admissions	PwD	Control group	*χ* ^2^	*P*-value
	(*N* = 23 327)	(*N* = 18 992)		
Fracture	4920 (21.1%)	2489 (13.1%)	665.469	**<0.001**
Urinary tract infection	4863 (20.8%)	1984 (10.4%)	1124.067	**<0.001**
Lower respiratory tract infection^a^	6636 (28.4%)	3642 (19.2%)	762.759	**<0.001**
Syncope and collapse	2013 (8.6%)	912 (4.8%)	317.985	**<0.001**
Cataract	2684 (11.5%)	3362 (17.7%)	270.921	**<0.001**

**ED**	**PwD**	**Control group**	***χ* ^2^**	***P*-value**
	(*N* = 18 953)	(*N* = 13 684)		

Fracture	2562 (13.5%)	1472 (10.8%)	190.375	**<0.001**
Head injury	1556 (8.2%)	591 (4.3%)	339.700	**<0.001**
Urinary tract infection	1198 (6.3%)	507 (3.7%)	215.727	**<0.001**
Soft tissue injury	962 (5.1%)	483 (3.5%)	106.395	**<0.001**
Lower respiratory tract infection^a^	1016 (5.4%)	506 (3.7%)	325.125	**<0.001**

a Lower respiratory tract infections included chest infection, pneumonia, bronchitis and bronchiolitis.

Bold *P*-value - significance <.001.

### Factors associated with hospital (re)admissions and ED visits

PwD had significantly higher odds of a hospital admission (odds ratio (OR) 1.79, 95% confidence interval (CI) 1.62–1.79, *P* < 0.001) and an ED visit (OR 1.35, 95% CI 1.29–1.42, *P* < 0.001), and significantly lower odds of a readmission (OR 0.60, 95% CI 0.57–0.63, *P* < 0.001) compared to controls. Table 3 presents factors associated with hospital admissions, re-admissions and ED visits in PwD. Female gender (*P* < 0.001) and living in less deprived areas (*P* < 0.001) were associated with lower odds of a hospital admission. Living in urban areas (*P* = 0.004) or transitioning to a care home (*P* < 0.001) was associated with increased likelihood of hospital admissions. Compared to married people, PwD who were widowed were more likely to be admitted to a hospital (*P* < 0.01).

**Table 3.** Binomial regressions assessing factors associated with hospital admissions and ED use in PwD

	Hospital admissions			Hospital readmissions			ED visits		
	OR	95% CI	*P*-value	OR	95% CI	*P*-value	OR	95% CI	*P*-value
Age	1.00	0.99–1.00	0.748	1.00	0.96–1.00	0.610	0.97	0.97–0.98	**< 0.001**
Gender									
Males		Ref		Ref			Ref		
Females	0.68	0.63–0.75	**< 0.001**	0.71	0.67–0.77	**< 0.001**	0.96	0.90–1.02	0.200
Deprivation	0.96	0.94–0.97	**< 0.001**	0.96	0.95–0.97	**< 0.001**	0.98	0.97–0.99	**< 0.001**
Living area									
Rural area		Ref		Ref			Ref		
Urban area	1.13	1.04–1.24	**0.004**	1.19	1.11–1.27	**< 0.001**	0.94	0.89–1.01	0.075
Marital status									
Married		Ref		Ref			Ref		
Single	0.94	0.71–1.26	0.692	0.86	0.73–1.01	0.061	1.09	0.95–1.26	0.203
Divorced/separated	1.51	0.82–2.78	0.186	1.36	0.98–1.87	0.063	1.01	0.79–1.28	0.966
Widowed	1.37	1.12–1.67	**0.002**	1.27	1.14–1.42	**< 0.001**	1.27	1.16–1.39	**< 0.001**
Care home residents	1.25	1.15–1.36	**< 0.001**	1.30	1.22–1.39	**< 0.001**	1.35	1.27–1.44	**< 0.001**

CI, confidence interval; OR, odds ratio.

Bold *P*-value - significance <.001.

Hospital readmissions for PwD could be predicted by living in urban instead of rural areas (*P* < 0.001), transitioning to a care home (*P* < 0.001) and being widowed instead of married (*P* < 0.001). Females (*P* < 0.001) and people living in less deprived areas (*P* < 0.001) were at lower risk of a hospital readmission.

Older age (*P* < 0.001) and living in less deprived areas (*P* < 0.001) were associated with lower odds of an ED visit. Transitioning to a care home (*P* < 0.001) and being widowed instead of married (*P* < 0.001) increased the likelihood of ED visits.

### Survival following hospital admissions and ED visits

At the time of censoring (31 December 2016), there were 13 514 (50.0%) deaths for PwD and 8067 (34.0%) deaths for the control group.

**Figure 1.**
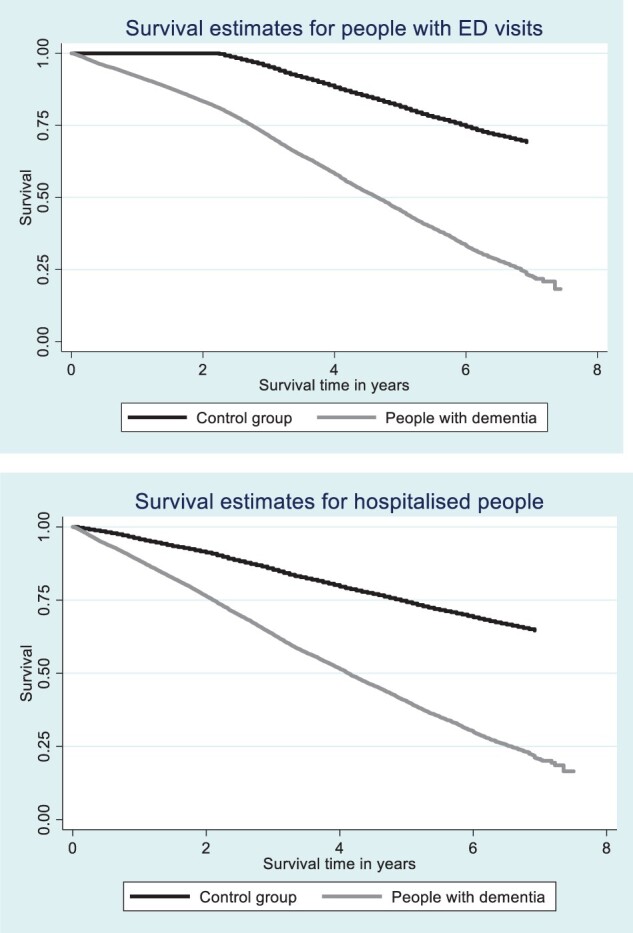
Survival time for people with hospital admissions and ED visits.

Mortality rates, after adjusting for age and gender, were higher for PwD who had been admitted to a hospital at least once (HR 3.074, 95% CI 2.98–3.172, *P* < 0.001, Figure 1) and for PwD with at least one ED visit (HR 3.961, 95% CI 3.812–4.117, *P* < 0.001) compared to controls.

The increasing number of hospital admissions over the study time frame was associated with higher mortality rates for people in the control group compared to people in the same group who had not been hospitalised (Table 4). On the other hand, PwD who were hospitalised for up to 40 times had lower mortality rates compared to PwD who had not been hospitalised. However, PwD who had been hospitalised between 41 and over 600 times were at higher risk of mortality compared to PwD who had not been hospitalised. Further breakdown of the number of hospital admissions was not possible because of small numbers of people as per HBS policy.

**Table 4.** Survival associated with hospital admissions, hospital stay in days and ED visits

	PwD					Control group				
	*N*	HR	CI	SE	*P*-value	HR	*N*	CI	SE	*P*-value
Number of hospital admissions										
0 times	3675	Ref					4842	Ref		
1–20 times	22 853	0.92	0.87–0.96	0.23	**<0.001**	1.43	18763	1.35–1.53	0.05	**<0.001**
21–40 times	253	0.76	0.63–0.95	0.80	**0.014**	1.87	271	1.55–2.24	0.18	**<0.001**
41–over 600 times	64	1.28	0.90–1.83	0.23	0.167	2.72	115	2.13–3.47	0.39	**<0.001**
Hospital stay										
0 days	5453	Ref					8219	Ref		
1–5 days	3855	0.81	0.75–0.86	0.03	**<0.001**	1.00	4333	0.91–1.09	0.04	0.962
6–10 days	3706	1.03	0.97–1.10	0.03	0.335	1.96	3263	1.81–2.12	0.08	**<0.001**
11–20 days	5474	1.25	1.18–1.32	0.04	**<0.001**	3.19	3685	2.98–3.42	0.11	**<0.001**
21–over 190 days	8357	1.48	1.41–1.56	0.04	**<0.001**	4.43	4491	4.16–4.72	0.14	**<0.001**
ED visits										
0 times	8008	Ref					10191	Ref		
1–2 times	8782	0.53	0.51–0.55	0.01	**<0.001**	0.63	7962	0.60–0.67	0.02	**<0.001**
3–4 times	4984	0.51	0.49–0.54	0.01	**<0.001**	0.74	3306	0.70–0.80	0.03	**<0.001**
5–10 times	4371	0.55	0.53–0.58	0.01	**<0.001**	0.80	2217	0.75–0.87	0.03	**<0.001**
11–20 times	628	0.53	0.47–0.60	0.03	**<0.001**	0.81	279	0.66–0.98	0.08	**0.031**
21–over 25 times	72	0.48	0.32–0.73	0.10	**0.001**	0.77	36	0.45–1.33	0.21	0.345

CI, confidence interval; HR, hazard ratio; SE, standard error.

Bold *P*-value - significance <.001.

PwD who spent up to 5 days in hospital had lower mortality rates compared to PwD who were not admitted to a hospital. However, prolonged hospital stays of 6 or more days increased mortality rates in both groups. The increasing number of ED visits was associated with lower mortality rates for PwD and the control group.

Within 6 months of a hospital admission, there were significantly more deaths in the dementia group (*n* = 4060, 30.04%) compared to the control group (*n* = 2101, 26.04%) [χ^2^(1) = 482.121, *P* < 0.001]. In addition, more PwD (*n* = 5029, 37.21%) died during a hospital admission compared to the control group (*n* = 4235, 52.50%) [χ^2^(2) = 1.700, 731, *P* < 0.001].

## Discussion

This study assessed the rates, causes and predictors of hospital admissions, readmissions and ED visits for PwD and a control group, as well as survival following this healthcare use. Similar to past research, this present study showed that more PwD had a hospital admission compared to a control group without dementia. On the other hand, the average number of hospital admissions for PwD was lower compared to the number of hospital admissions in the control group. However, PwD stayed in the hospital for more days compared to controls.^1^^,^^3^^,^^7^ A possible explanation is that PwD have more comorbidities.^7^ The prolonged hospital stay for PwD could also explain the reason for fewer hospital admissions and ED visits compared to controls. In terms of the time of death, more PwD died during or following a hospital admission compared to people in the control group. This finding could mean that PwD are hospitalised when they are very ill. The prolonged hospital stays for PwD are in line with past research^22^ and could be explained by the disease severity, with PwD being hospitalised when they are very ill compared to people without dementia or because PwD are more likely to experience delirium, resulting in a longer recovery.^5^

PwD were also more likely to have an ED visit, possibly because they have more medical comorbidities. In our study, the main reasons for hospital admissions and ED visits aligned with those from past research. PwD were more often admitted to a hospital or ED because of a fracture, a urinary tract infection or a lower respiratory infection.^2^,^6–8^^,12^ However, PwD were hospitalised for cataract less often compared to controls. PwD are less likely to undergo surgery for cataract^23^ and thus are less likely to be hospitalised for same. PwD in a care home had a higher odds of ED visits, hospital admissions and readmissions compared to controls. Past research has shown increased number of ED visits for PwD who transitioned to a nursing home.^5^ This most likely reflects the number of co-morbidities and complex care needs of care home residents with dementia. Only one study has shown that PwD in the community were admitted more frequently to a hospital compared to PwD who transitioned in care homes, but this study included fewer participants (*n* = 1700), and PwD were followed up for three months only.^24^ A healthcare professionals’ survey carried out in 2016 highlighted that admission to hospital from a care home was deemed inappropriate in 54.6% of cases, albeit not all of the patients had dementia. Recurring themes were uncertainty around services available to care homes and anticipatory care planning. The lack of consensus suggested that the concepts surrounding inappropriate hospital admission are not shared by staff that provide care for care home residents.^25^

In contrast to past research,^11^ widowed PwD were more likely to be hospitalised and to visit an ED compared to married people. The presence of a partner as a primary caregiver is often associated with increased likelihood of receiving treatment, such as medication, compared to people who live without a caregiver.^26^ Nonetheless, further research has shown, people living alone, such as widowed people, are more likely to use health services at home^8^^,^^27^ and to be admitted to hospitals or transition to a care home.^27–29^ The increased likelihood of widowed people to receive medical care may be due to the absence of informal care at home. Another possible explanation is that married PwD are encouraged by their partner to adopt a healthier lifestyle, including regular physical exercise and a balanced diet.^29^

Living in less deprived and rural areas was associated with fewer hospital admissions. This could be due to different health and care services in urban and rural areas. In contrast to past findings,^7^ PwD had lower mortality rates with up to 40 admissions to a hospital or an ED visit. This may reflect good care provided to PwD and it shows the necessity of providing good healthcare for PwD. Our results showed that PwD who had over 41 hospital admissions were at higher risk of death compared to people who had not been hospitalised; however, the small number of people in this case is a limitation of this study. These mortality rates may reflect disease severity in PwD. As patients progress from mild to moderate to severe dementia, their healthcare needs increase, often leading to a higher number of hospital admissions. The PwD may benefit more from hospital admission at an early stage of their disease rather than towards the end of life.

One important limitation of this study concerns the identification of PwD. Dementia cases were initially identified through dementia medication dispension. However, dementia was reported as a cause for hospitalisation or death for 1427 people in the control group. Although these people were transferred to the dementia group, it is possible that our control still includes some PwD who did not receive dementia medication, were alive by December 2016, and were either not hospitalised or dementia was not recorded during their hospitalisation. In addition, transferring 1427 people from the control group to the dementia group resulted in two groups that were not age- and gender-matched; however, we controlled for age and gender in our main analysis. Moreover, using dementia medication as a proxy for a dementia diagnosis means that we identified mostly people with Alzheimer’s disease, mixed dementia and Lewy Body dementia. Another limitation of this study concerns the marital status, which was recorded only for people who had been hospitalised and was never updated following subsequent hospitalisations.

To conclude, our results indicated greater healthcare use by PwD compared to people without dementia that could help planning for healthcare services. PwD were at less risk of death following an ED visit for their first 40 hospital admissions. Therefore, policies should focus on developing equally efficient care services to PwD, especially for PwD who are widowed, live alone or lack mobility, in order to prolong survival, but also to avoid hospital admissions towards the end of life if possible.

## Data Availability

Data can be accessed through the Honest Broker Service (HBS) within the Business Services Organisation Northern Ireland (BSO).
